# Deciphering the sequential changes of monocytes/macrophages in the progression of IDD with longitudinal approach using single-cell transcriptome

**DOI:** 10.3389/fimmu.2023.1090637

**Published:** 2023-02-01

**Authors:** Weihang Li, Yingjing Zhao, Yongchun Wang, Zhijian He, Linyuan Zhang, Bin Yuan, Chengfei Li, Zhuojing Luo, Bo Gao, Ming Yan

**Affiliations:** ^1^ Department of Orthopedic Surgery, Xijing Hospital, Air Force Medical University, Xi’an, China; ^2^ Department of Critical Care Medicine, Nanjing First Hospital, Nanjing Medical University, Nanjing, Jiangsu, China; ^3^ Department of Aerospace Medical Training, School of Aerospace Medicine, Air Force Medical University, Xi’an, China; ^4^ Department of Sports Teaching and Research, Lanzhou University, Lanzhou, China; ^5^ Department of Nursing, Air Force Medical University, Xi’an, China; ^6^ Department of Spine Surgery, Daxing Hospital, Xi’an, Shaanxi, China

**Keywords:** intervertebral disk degeneration, monocytes/macrophage subtypes evolution, oxidative stress, trajectory lineage analysis, single-cell transcriptome landscape, longitudinal approach

## Abstract

Intervertebral disk degeneration (IDD) is a chronic inflammatory disease with intricate connections between immune infiltration and oxidative stress (OS). Complex cell niches exist in degenerative intervertebral disk (IVD) and interact with each other and regulate the disk homeostasis together. However, few studies have used longitudinal approach to describe the immune response of IDD progression. Here, we conducted conjoint analysis of bulk-RNA sequencing and single-cell sequencing, together with a series of techniques like weighted gene co-expression network analysis (WGCNA), immune infiltration analysis, and differential analysis, to systematically decipher the difference in OS-related functions of different cell populations within degenerative IVD tissues, and further depicted the longitudinal alterations of immune cells, especially monocytes/macrophages in the progression of IDD. The OS-related genes CYP1A1, MMP1, CCND1, and NQO1 are highly expressed and might be diagnostic biomarkers for the progression of IDD. Further landscape of IVD microenvironment showed distinct changes in cell proportions and characteristics at late degeneration compared to early degeneration of IDD. Monocytes/macrophages were classified into five distinct subpopulations with different roles. The trajectory lineage analysis revealed transcriptome alterations from effector monocytes/macrophages and regulatory macrophages to other subtypes during the evolution process and identified monocytes/macrophage subpopulations that had rapidly experienced the activation of inflammatory or anti-inflammatory responses. This study further proposed that personalized therapeutic strategies are needed to be formulated based on specific monocyte/macrophage subtypes and degenerative stages of IDD.

## Introduction

Low back pain (LBP) has continuously been the major cause of disability in human adults, affecting almost 80% of the population worldwide, leading to heavy socioeconomic burden (1, 2). There are lots of triggers of LBP, and intervertebral disk degeneration (IDD) is considered as one of the common reasons (3). IDD progression is a commonly seen musculoskeletal disorder, accompanied by gradual structure alteration with several metabolic homeostasis process, spinal stenosis, disk herniation, lumbar spondylolisthesis, spinal segmental instability, nerve root compression, etc. (4, 5). Consequently, a better understanding of the potential pathological mechanisms has continuously been a research hotspot, which may help to promote the knowledge of IDD progression and finally find novel biological treatments.

Increasing evidence has reported that despite multifactorial etiology like loading changes, smoking, senescence, and poor nutrient supply, genetic factor is the essential risk of IDD, which accounts for more than 70% of the risks (2). Meanwhile, as the largest avascular organ within body, IVD consists of the middle nucleus pulposus (NP), surrounding annulus fibrosis (AF), and cartilage endplate (CEP) on the inferior and superior sides. This special structure makes it an immune-privilege organ and avoids infiltration by immune and inflammatory factors (6, 7). When IDD occurs, the blood vessels infiltrate and inflammatory factors aggregate at the IVD tissue, destroying the homeostasis of IVD (8).

Redox homeostasis is a pivotal process for the physiological maintenance of many cellular activities; the dysregulation of redox homeostasis is tightly related to diversity pathological conditions that influence human health, including different kinds of neoplasms and degenerative skeletal disease (9–13). Oxidative stress (OS) is regarded as the imbalanced state of redox homeostasis with higher reactive oxygen species (ROS) production; OS also plays essential roles in the progression of IDD (12, 14). It has been elucidated that OS could induce autophagy, apoptosis, and calcification of CEP chondrocytes (15–17). Existing studies also reported that the excessive accumulation of ROS would cause OS reaction and injure the normal functions and integrity of NP cells, thereby triggering inflammatory reactions and promoting IDD progression (18, 19).

Recent advanced techniques in single-cell RNA sequencing (scRNA-seq) have provided powerful algorithm for exploring and analyzing the interactive roles and functional heterogeneity of different cell populations (20–22). These methods have been widely performed in different fields like transcriptomic atlas construction and novel populations identification, which have revolutionized studies of gene expression (23, 24). Existing studies have already depicted the transcriptomic atlas for the IVD tissue based on scRNA-seq, which discovered and defined different or new cell subpopulations, providing comprehensive interpretations and novel insights in cellular heterogeneity of IVD, including physiological and degenerative states (25–29).

Immune cells, especially monocytes/macrophages, are reported to produce ROS and promote the onset of OS, causing severe metabolic disorders and even cell death; ROS could also activate transcriptional factors and pro-inflammatory factors and further accelerate inflammation reactions (30). Consequently, identifying functional OS-related immune cells can increase our understanding of OS mechanisms and provide novel antioxidative avenues in the treatment of IDD. However, due to the intrinsic limitations that most scRNA studies have in providing cell atlas in a specific tissue without lucubrating the cell subtypes, the current available transcriptomic analyses of immune cells are from cross-sectional studies, with rare longitudinal description of the immune response. This study performed conjoint analysis of bulk RNA-seq and scRNA-seq to analyze OS functions in different cell populations from early to late degenerative IDD patients. This is the first such landscape to depict the longitudinal alterations of NP cells and monocytes/macrophages heterogeneity in the progression of IDD. Importantly, we revealed the evolution process of monocytes/macrophages, identified different subtypes based on their unique gene expression patterns, and described their temporal transcriptome changes from early to late degenerative IVD tissue. Intriguingly, the highly dynamic populations, effector monocyte/macrophages, and regulatory macrophages were mainly enriched in early degenerative stage, while the homeostatic and activated tissue macrophages were more like terminal differentiated populations that increased during disease progression, which could provide reference for monocyte/macrophage targeted therapy at different stages of IDD.

## Materials and methods

### Data collection and preprocessing

Microarray datasets were retrieved and downloaded from Gene Expression Omnibus (GEO, https://www.ncbi.nlm.nih.gov/geo/) database. Bulk RNA-seq data GSE70362 was obtained for OS functional analysis between different degenerative stages, which contained 48 IDD patients with Pfirrmann grade from I to V; grade <3 was considered as early degenerative, and grade ≥3 was late degenerative stage (31). GSE147383 was applied to evaluate OS functional levels and immune infiltration situation and construct weighted gene co-expression network analysis (WGCNA) of degenerative IVD tissues (32). Then, GSE165722 series data were used for scRNA-seq analysis to analyze the OS functional levels between different cell populations and depict transcriptome atlas during different degenerative stages, which included eight patients with degenerative stages from II to V. Only NP tissues were harvested, following a standard surgical protocol. No AF tissues and blood were contaminated the into NP tissue upon collection (25). This study included multiple datasets for analysis to avoid the bias from the same dataset and make the results more convincing.

### Identification of differentially expressed OS genes

According to Pfirrmann grade, bulk RNA-seq data (GSE70362) totally contained 16 early degenerative samples and 32 late degenerative samples. This study divided patients into two groups and further screened differentially expressed genes (DEGs) with “limma” package (3.50.0) in R, followed by a cutoff threshold with adjusted p-value < 0.05 and |FC| (fold change) > 1.5, which was considered as statistically significant. To identify DEOSGs, we further extracted 824 OS-associated protein domains from GeneCards database (https://www.genecards.org/) with a relevance score ≥ 7 (see in **Supplementary Table S1**); then, DEOSGs were screened by taking the intersection of DEGs and OS-related genes to generate a more accurate result, based on Venn plot analysis (“VennDiagram” package in R, 1.7.1).

### Immune infiltration analysis

The proportions of the 22 types of immune cells in IVD tissues were estimated using “CIBERSORT” algorithm (33). The proportions and contents of immune cell members from mixed IVD tissues were assessed through gene expression profiles, followed by correlation heatmap and differential expression patterns between different groups. Immune infiltration analysis demands strict requirements on data; only high contents of estimated immune cells are appropriate for analysis. After evaluation of the immune cells components, GSE147383 possessed the highest quality, which was conducted for immune infiltration to analyze the potential existing immune cells, which contained eight samples.

### Construction of weighted gene co-expression network and immune-related DEOSGs identification

The top 5,000 most variable genes were included for WGCNA (“WGCNA” package in R, 1.71) construction, based on their median absolute deviation (MAD) values according to official introduction (34). Hierarchical clustering analysis was performed to detect outliers of these tissues, and the soft threshold power (β) was calculated to build a scale-free network. Weighted gene co-expression network was constructed based on β-value; then, co-expression modules were identified according to the heterogeneity of different genes. The relationships between each module and differentially infiltrating immune cells were then calculated and visualized. As Li et al. described, the module with the highest correlation coefficient was considered as the candidate module, which was compared horizontally to discover the most essential genes targeting specific immune cell (35). The intersection of DEGs, OS-related genes, and genes in the interested module was considered as the most related genes (hub genes) for the progression of IDD.

### Hub genes validation by principal component analysis and gene expression detection

Principal component analysis (PCA) was used to reduce the dimension of these hub genes from high dimension into three dimensions (PC1, PC2, and PC3), to observe the separating ability between early and late degenerative IVD tissues, based on “prcomp” and “princomp” functions in R. The results were visualized in three-dimensional coordinate system (“scatterplot3d,” “ggplot2,” and “rgl” packages in R, 3.3.5). Human primary NP cells were obtained from iCellbioscience (HUM-iCell-s012) and were maintained in Dulbecco’s modified Eagle’s medium (DMEM)/F12 (1:1) (DF12; Gibco, Grand Island, NY, United States) with 10 fetal bovine serum (FBS; Invitrogen, Carlsbad, CA, United States) and 1% antibiotics (penicillin/streptomycin) (Gibco) in an incubator at 5% CO_2_ and 37°C (36, 37). Tert-butyl hydroperoxide (TBHP) was purchased from Sigma-Aldrich (St. Louis, MO, United States), to trigger oxidative stress and thus simulate high-ROS environment, which is a stable form of hydrogen peroxide and widely used as an *in vitro* model to induce extracellular matrix (ECM) degeneration and the apoptosis of NP cells (38). To establish the apoptosis and degenerative model of NP cells, complete culture media with different concentrations (0, 50, and 100 μM) of TBHP were added for 24 h (38). Then, the expression levels of those hub genes under different conditions of degenerative NP cells were detected by quantitative real-time PCR (qRT-PCR). The primers of corresponding genes were listed as follows (5–3′): CCND1, (F) GATGCCAACCTCCTCAACGA and (R) ACTTCTGTTCCTCGCAGACC; CYP1A1, (F) CAAGGGGCGTTGTGTCTTTG and (R) GTCGATAGCACCATCAGGGG; MMP1, (F) TGTGGTGTCTCACAGCTTCC and (R) CGCTTTTCAACTTGCCTCCC; and NQO1, (F) TTTGGAGTCCCTGCCATTCT and (R) TTGCAGAGAGTACATGGAGCC.

### Single-cell RNA data integration and processing

The raw scRNA-seq data of NP tissues (Pfirrmann grade II–V) stages were retrieved and obtained (based on GSE165722). After the whole data were read and presented in R, filtering criteria were followed by rigorous procedure, and low-quality cells were removed as follows: cells had fewer than 200 expressed genes and mitochondria UMI counts rate >20%.

After quality control, we created Seurat objects from scRNA-seq data based on each tissue according to “Seurat” package in R (4.1.0) (39). Then, RPCA algorithm wrapped in “Seurat” package was applied to integrate the expression matrix of each tissue and correct batch effects among different tissues; “FindIntegrationAnchors” function was used to merge samples with common anchors among variables (dims = 1:30, k.anchors=10). Then, the integrated matrix was followed by cell normalization and scale. In brief, the top 2,000 highly variable features were analyzed after normalization, which were selected for downstream analysis. Then, PCA was constructed using “RunPCA” function based on the scaled data with the top 2,000 highly variable genes, and the top 30 principles were further chosen for uniform manifold approximation and projection (UMAP) and t-distributed stochastic neighbor embedding (tSNE) dimensional reduction. The unsupervised cells were clustered together by “FindClusters” and “FindNeighbors” functions based on the top 30 PCA principles, to cluster the similar types of cell populations together, with resolution setting as 0.3. The marker genes of each cell cluster were detected by “FindAllMarkers” function with the following criteria: min.pct > 0.1, logfc.threshold > 0.25 and p-value < 0.01. Immune cells were further extracted out and conducted for subsets subdivided, followed by re-tSNE and re-UMAP dimensional reduction.

### GSVA algorithm assessed the OS functional levels

To evaluate the differences in OS-related functions in different degenerative grade samples, we retrieved and downloaded GO BP terms from the MsigDB database (Molecular Signatures Database, https://www.gsea-msigdb.org/gsea/msigdb/) to screen the required OS-related functional items (40). “Single sample GSEA (ssGSEA)” algorithm was conducted in R (“GSVA” package, 1.42.0) to calculate functional scores in each sample. Higher scores indicated higher levels of the functional item in each sample. We obtained the following items to fully assess the OS-related functions in IVD tissues: “GO_RESPONSE_TO_OXIDATIVE STRESS,” “GO_REGULATION_OF_RESPONSE_TO_OXIDATIVE_STRESS,” “GO_CELL_DEATH_IN_RESPONSE_TO_OXIDATIVE_STRESS,” and “GO_CELLULAR_OXIDANT_DETOXIFICATION,” as Yu et al. described (41). OS-related scores of different cell populations were calculated to evaluate these OS functions.

### Differentially gene expression analysis

“FindMarkers” function was applied to detect DEGs among different groups or cell populations within scRNA-seq data, followed by the following criteria: Wilcox rank sum test algorithm, min.pct > 0.1, logfc.threshold > 0.25, p-value < 0.01, and only.pos = TRUE.

### Functional and pathway enrichment analysis of DEGs and sub-populations

DEGs and marker genes of each cell population were analyzed by R (“clusterProfiler” package, 4.2.1) and DAVID database (https://david.ncifcrf.gov/ ) to get understanding of functional annotations and interpretations, including Gene Ontology (GO) and Kyoto Encyclopedia Genes and Genomes (KEGG) results. p-value < 0.05 and false discovery rate (FDR) < 0.25 were considered as significantly enriched. For each cell population, the top 50 genes (prioritized by fold change when comparing each cluster with the rest) were conducted to the enrichment analysis, and ontology terms with near-duplicated terms were eliminated using custom script. The exclusion criteria of GO terms were “POSITIVE,” “NEGATIVE,” “EOSINOPHIL,” “T_HELPER,” or “T_CELL,” as Lee described (42).

### Monocytes/macrophages evolvement analysis

To analyze the evolvement procedure of monocytes/macrophages, this study applied CytoTRACE analysis to decode the relative evolutionary states of different subtypes, which was one of the computational methods for depicting the states of cell fate without any prior information (43). The monocytes/macrophages evolvement process was thus analyzed by CytoTRACE with the default parameter.

### Differentiation trajectory of monocytes/macrophages by pseudotime analysis

The trajectory analysis of monocytes/macrophages was performed by Monocle2 algorithm (44). After the calculation of size factor and dispersions estimation, DEGs along the trajectory branch were further identified. Seurat clustering results and raw expression counts matrix of cells were prepared before monocle analysis, followed by “DDR-Tree” method and other default parameters to reduce dimensions. Based on pseudotime results, evolvement procedure of monocytes/macrophages were conducted by branch expression analysis modeling (BEAM) analysis.

### Cell–cell interaction analysis

To investigate cellular interactive communications among different cell populations, this study conducted CellPhoneDB (based on Python 3.7) and CellChat to get a systematic understanding for cell–cell communication molecules (45, 46). CellPhoneDB and CellChat were public databases for ligands, receptors, and their interactions, where the membrane and secreted and peripheral proteins of the clusters were annotated. The normalized cell expression matrix was prepared by Seurat normalization; the significant mean and cell communication significance were calculated based on each interactive role, setting as p-value < 0.05.

### Transcription factor network inference

To further decode the gene regulatory network of different monocyte/macrophage subtypes, we performed single cell regulatory network inference and clustering (SCENIC) algorithm to identify specific regulons involved in different cell subpopulations (based on pySCENIC and R-SCENIC) (47). After extraction of count expression matrix, the transcription factor (TF) activities (AUCell) for each cell were calculated through motif library (version “mc9nr”), and the TF regulation strength was assessed using the 20,000 motifs database according to RcisTarget and GRNboost.

### Statistical analysis

Data statistical analysis and visualization in this study was achieved by R (version 4.1.3, based on different packages and algorithms mentioned above) and GraphPad Prism (version 8.3.0). One-way ANOVA analysis was chosen flexibly to compare three or more groups, and Student’s t-test was used for statistical analysis between two groups. The difference of p< 0.05 was considered as statistically significant.

## Results

### Oxidative stress levels increased gradually with degenerative stages

The human body is always within a balanced state between the oxidant and antioxidant system. The imbalance refers to overexpression of ROS and reduction in antioxidant capacity. Thus, we compared and analyzed the OS functional levels of IVD tissues under different degenerative stages, based on GSE70362 and GSE147383.

**Figure 1:**
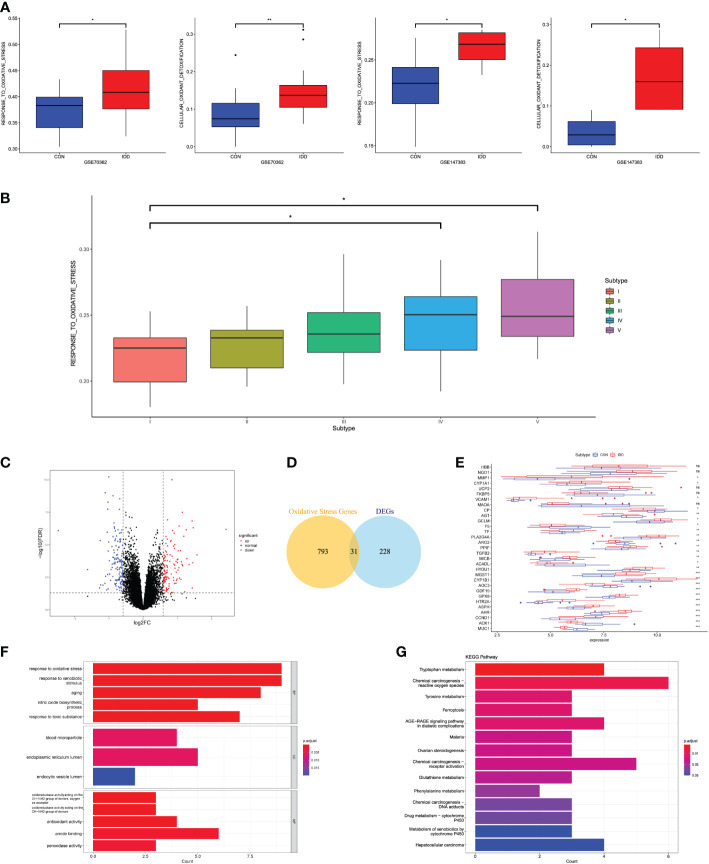
**(A)** Differences in cellular oxidative stress response in GSE70362 and GSE147383, respectively. **(B)** OS-related functional scores in each degenerative stage. The higher the score, the more active function. **(C)** Volcano plot of the identified DEGs. **(D)** Venn diagram indicating 31 genes were commonly expressed as DEOSGs. **(E)** The expression situations of these 31 DEOSGs in CON and IDD groups. **(F, G)** Representative analysis of GO, KEGG categories showing different functions of the screened 31 DEOSGs. “CON” group represented “early degenerative patients,” and “IDD” group represented “late degenerative patients”, *p < 0.05, **p < 0.01, ***p < 0.0001, ns: none significance, which were the same as below.

According to the relevant functional items retrieved from MsigDB database, we performed GSVA algorithm to score each different degenerative IVD tissue from Pfirrmann grades I–V. We evaluated the OS-related functions like “GO_RESPONSE_TO_OXIDATIVE STRESS,” “GO_REGULATION_OF_RESPONSE_TO_OXIDATIVE_STRESS,” and “GO_CELL_DEATH_IN_RESPNSE_TO_OXIDATIVE_STRESS.” Results illustrated that the OS-related levels in severe degenerative IVD tissues were significantly higher than that in early degenerative tissues among different datasets (**Figure 1A**). Then, we analyzed the OS-related scores in each degenerative stage. Results revealed that the OS levels elevated with the degenerative stage, and OS levels in grades IV and V were significantly higher than that in grade I, which demonstrated the close correlations of OS reactions with the progression of IDD, as shown in **Figure 1B**.

### Identifying DEOSGs related with the OS functions during IDD progression

This study divided the total gene expression matrix of GSE70362 into two groups (early and late degenerative group), followed by linear model fitting and Bayes detection. With cutoff criteria setting as adjusted p < 0.05 and |FC| > 1.5, 259 DEGs were totally screened, with 124 upregulated and 135 downregulated genes. The volcano plot visualized the distribution of these DEGs between early and late degenerative IVD tissues (**Figure 1C**). Then, the DEOSGs were further identified by the intersection of 259 DEGs and 824 OS-related genes. Altogether, 31 genes were filtered as DEOSGs, which were associated with the progression of IDD (**Figure 1D**). We further analyzed the gene expression values of each DEOSG, as shown in **Figure 1E**.

Functional enrichment analysis was further applied to understand the roles in the progression of IDD. GO analysis suggested that the upregulated DEOSGs were primarily correlated with biological functions like “response to oxidative stress,” “response to xenobiotic stimulus,” “response to toxic substance,” “peroxidase activity,” “oxidoreductase activity,” and “aging”; besides, the cellular components were mainly located in the endoplasmic reticulum lumen (**Figure 1F**). These results indicated the essential roles of OS in the progression of IDD. The KEGG pathway illustrated that these DEOSGs were mainly involved in “chemical carcinogenesis production-ROS,” “DNA adducts,” “receptor activation,” “ferroptosis,” “tryptophan and tyrosine metabolism,” and “metabolism of xenobiotics by cytochrome P450” (**Figure 1G**).

### Immune-infiltrating cell analysis

**Figure 2:**
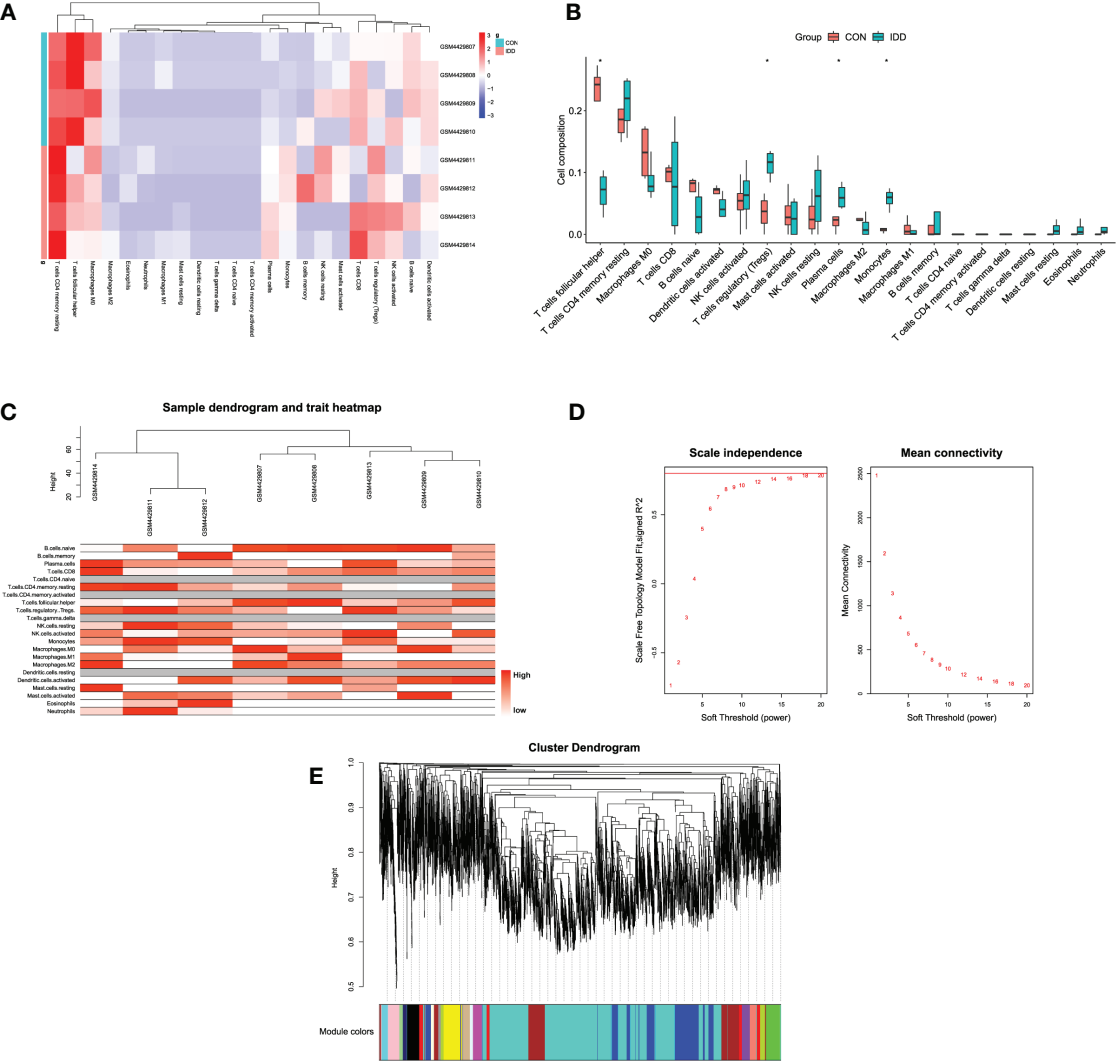
**(A)** Heatmap displaying the distribution of 22 types of immune cells in different groups. **(B)** The relationships of immune infiltration levels between different groups. **(C)** Cluster dendrogram of samples to detect outliers. The color is from white to red; the redder color represented higher contents of immune cells. **(D)** Selection of the soft threshold power value. The left panel represents scale-free model fit index; the right panel represents the mean connectivity of these values. **(E)** Cluster dendrogram of genes enriched based on dissimilarity measure and assignment modules.

To understand the compositions of immune cells between normal and degenerative IVD tissues, this study applied “CIBERSORT” method from GSE147383 to discover the correlated immune cells involved in the progression of IDD. The “CIBERSORT” method was designed to estimate the relative proportion of different types of immune cells *via* deconvolution algorithm. **Figure 2A** and **Supplementary Figures S1E, F** displayed the enrichment fraction of immune-infiltrating cells in IVD tissue. Results illustrated that T-cells regulatory (Tregs), plasma cells, and monocytes/macrophages were significantly activated and T-cell follicular helper (Tfhs) were significantly inhibited in late degenerative IVD tissues, as shown in **Figure 2B**, which suggested the potential regulatory roles of these immune cells in the progression of IDD.

### Construction of co-expression network and identification of interested modules

**Figure 3:**
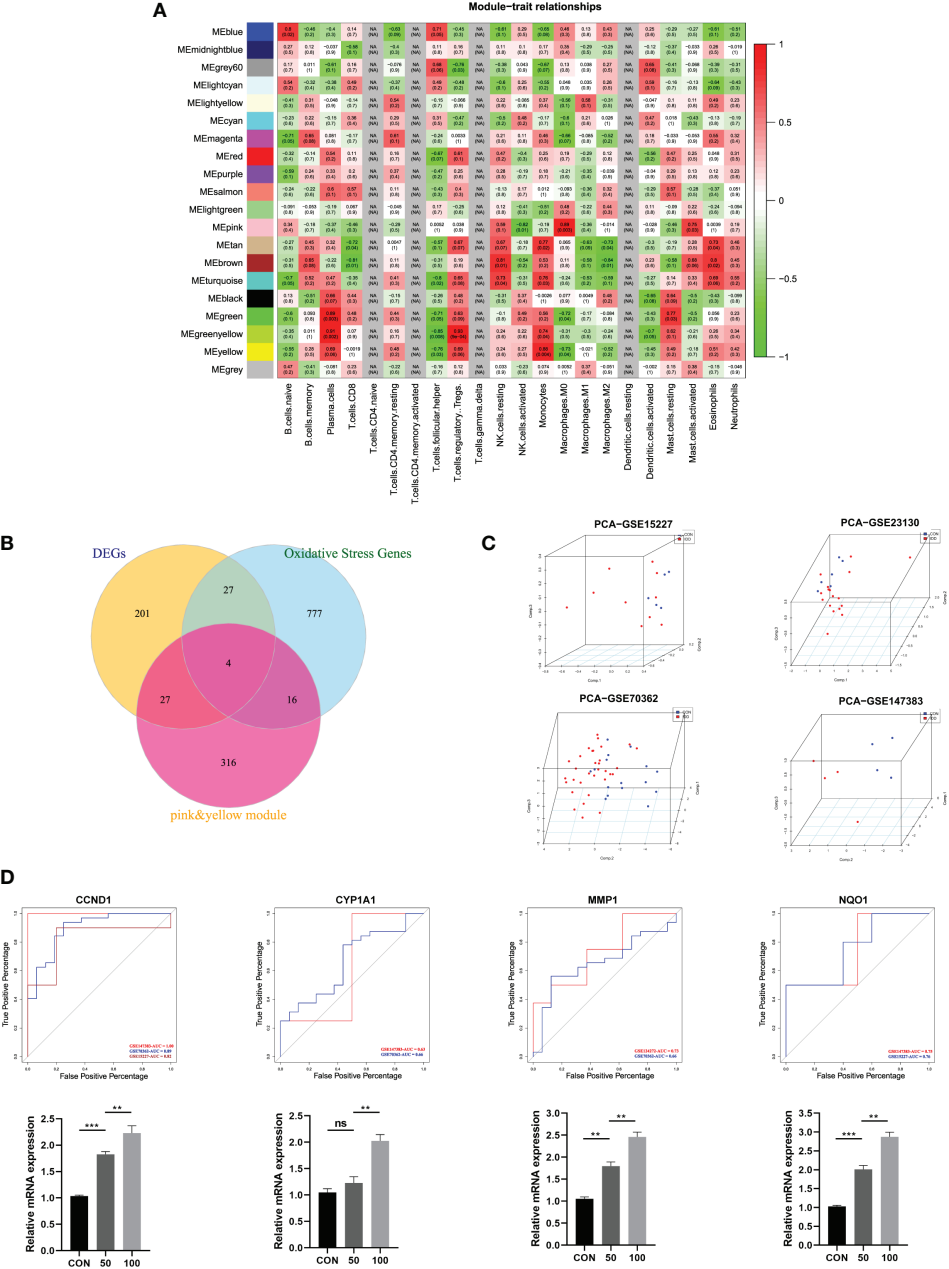
**(A)** Module–trait relationships between different clustered modules and immune infiltration cells. Green represents negative correlation, and red represents positive correlation; the darker colors indicated higher weights. Each square also contained corresponding correlation (first line) and p-value (second line), which were consistent with the shade of the color. **(B)** Venn diagram showing the hub genes among three parts. **(C)** 3D scatter plot after PCA dimensional reduction in these hub genes in different datasets. **(D)** ROC curve analysis for predicting IDD in different datasets; qRT-PCR was used to detect the mRNA expression of CCND1, CYP1A1, MMP1, NQO1 in human NP cells treated with TBHP for different concentrations.

After samples heterogeneity detection, all samples were included in the WGCNA workflow, together with their immune cells’ information (**Figure 2C**). Soft threshold power was determined as 18 based on the scale-free network construction (**Figure 2D**). Then, hierarchical clustering tree analysis was performed based on the mutual genes’ co-expression; altogether, 20 modules were generated, and each module had unique gene expression patterns (**Figure 2E**). Furthermore, the relationships between modules and clinical traits (infiltrating immune cells) was demonstrated in **Figure 3A**. The genes in these generated modules are associated with the progression of IDD, the color depth, and correlation coefficient, and the p-value is correlated with the weights of IDD occurrence. Since the immune infiltration analysis algorithm was developed based on gene expression, the real monocytes/macrophages subtypes could not be analyzed accurately. Results discovered several interested modules like pink, yellow, and green yellow modules targeting macrophages M0 (Cor = 0.89, p = 0.003), monocytes (Cor = 0.88, p = 0.004), plasma cells (Cor = 0.91, p = 0.002), and Tregs (Cor = 0.93, p = 9e−04), respectively. Pink and yellow modules were further merged together as essential modules of monocytes/macrophages and conducted for further analysis to avoid the loss of gene information.

### Identification of differentially expressed immune-related oxidative stress genes and hub genes validation

The differentially expressed immune-related OS genes, namely, hub genes, were finally identified by Venn plot analysis; the intersection of the three parts included screened 259 DEGs, 824 OS-related genes, and genes in pink and yellow modules. Totally, four genes were obtained: CYP1A1, MMP1, CCND1, and NQO1 (**Figure 3B**).

Then, the robustness of these four genes was validated by PCA dimensional reduction analysis among different databases. PCA was performed to reduce the dimension of these hub genes into three principal components PC1, PC2, and PC3, where each dot plot indicated each sample in the three-dimensional coordinate system. Based on the gene expression matrix of each database, these dot plots showed significantly distinguishment ability in spatial distribution (**Figure 3C**).

Based on the four genes identified in this study, we further conducted ROC curve analysis to assess the predictive ability of each feature to observe their diagnostic ability of IDD. As shown in **Figure 3D**, results indicated high area under curve (AUC) value of each gene among different datasets. The AUC of CYP1A1 was 0.63, 0.66; AUC of MMP1 was 0.73, 0.66; AUC of CCND1 was 1.00, 0.89, 0.82; and AUC of NQO1 was 0.75, 0.76, respectively. Meanwhile, the PCR results further validated that these four hub genes were significantly upregulated in degenerative NP cells with the concentration of TBHP, which further proved the essential roles of these genes in the progression of IDD. Consequently, these four genes were determined as hub genes in this study, which had the most connections with the progression of IDD.

### Single-cell profiles landscape revealed the immune cellular heterogeneity within different degenerative stages of IVD tissues

**Figure 4:**
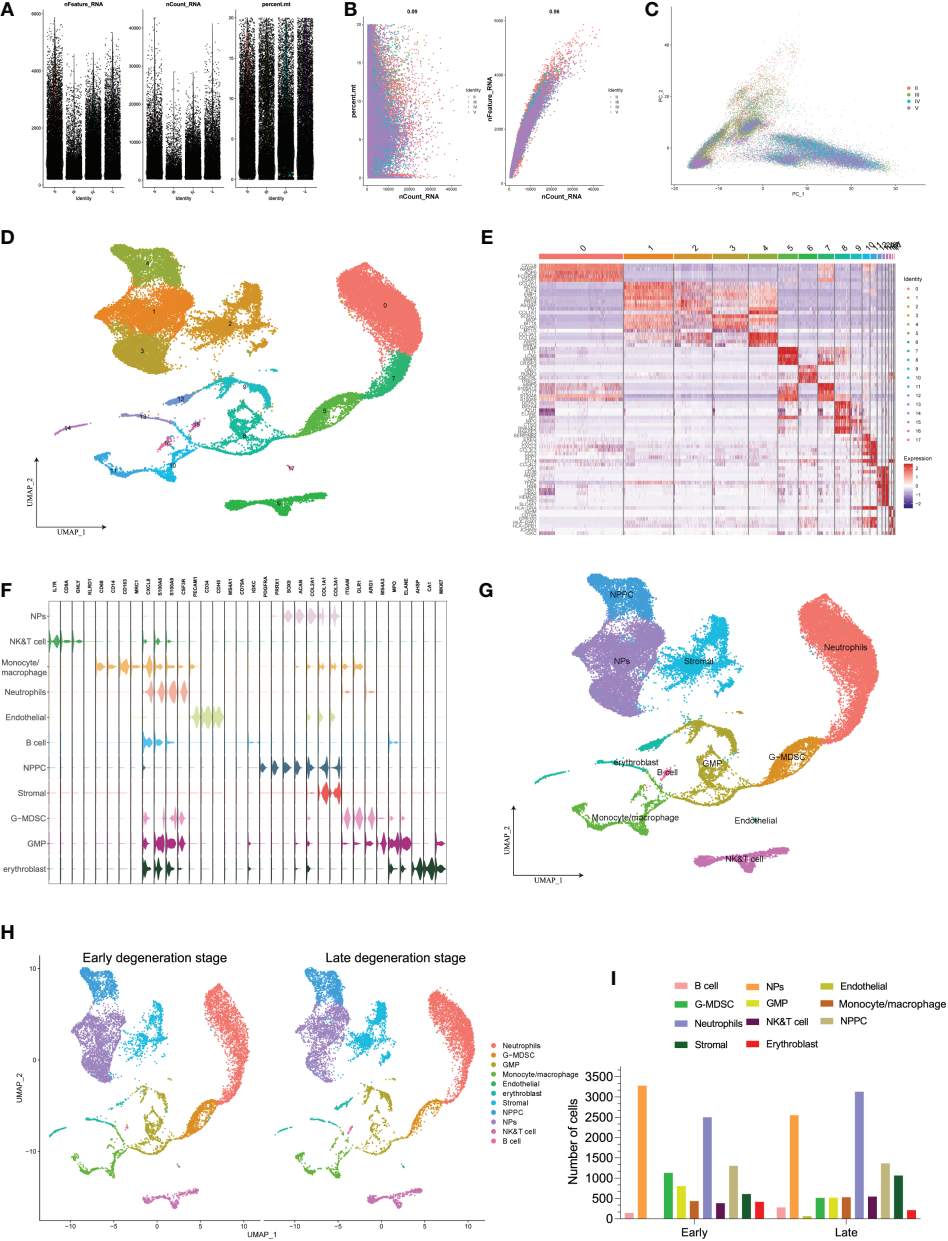
**(A)** Scatter plot after quality control and filtering of cells. **(B)** The left panel indicates that the mitochondria were the same with sequencing depth; the right panel suggests that the numbers of the detected genes were significantly related to the sequencing depth. **(C)** PCA scatter plot displayed the dot distribution after sample integration. **(D)** UMAP visualization of degenerative IVD, which identified 17 cell clusters. **(E)** The heatmap displays the top 5 genes of the identified markers genes of each cluster. **(F)** The violin plot shows the mean expression of the selected marker genes used for annotation in each cell cluster. **(G)** UMAP visualization after annotation for each cell cluster. **(H)** UMAP visualization of cell populations distribution between early and late degeneration stage. **(I)** The numbers of each cell type in different degeneration stage.

In the above analysis, we found that some immune cells like Tregs, plasma cells, and monocytes/macrophages were upregulated aberrantly in IDD groups. Therefore, we further depicted immune cells heterogeneity atlas within IVD tissues at single-cell level (based on GSE165722). After rigorous quality control workflow to eliminate cells with low gene detection (<200 genes) and high mitochondrial gene content (>20%), a total of 42,960 cells with 18,142 expressed genes were included for subsequent analysis (**Figure 4A**). The number of the detected genes was significantly correlated with the sequencing depth, and mitochondria UMI rate of each cell was lower than 20% (**Figure 4B**). The variance analysis revealed the top 2,000 highly variable genes of each sample, and PCA method was performed to identify available dimensions based on these principals; dot plots and heatmap also displayed the significant correlated genes for cell clustering (**Supplementary Figures S1A–C**). After elimination of different samples by RPCA integration algorithm, PCA and UMAP scatter plot suggested a high overlap of these cells, whether by grades or patients, indicating good integration result (**Figure 4C** and **Supplementary Figure S1D**). Then, the top 50 PCs were calculated based on the top 2,000 genes, and the top 30 PCs with the lowest p-values were chosen for subsequent dimensional reduction analysis.

Unsupervised dimensional reduction method was performed for cell subtype clustering by UMAP algorithm; totally, 18 cell clusters were generated in the NP tissue, and according to marker genes detection of each cluster, different cell populations showed high heterogeneity with each other (**Figure 4D**). The detailed types of cell clusters were referenced by singleR, combined with the existing literatures and databases (48). The heterogeneity of different cell clusters was classified by gene expression (**Figure 4E**), and 11 clear separations of cell subtypes were annotated, including six types of immune cells and the rest types of NP-related cells. The representative gene expression patterns of specific markers are shown in **Figures 4F, G**: the core genes encoding anabolic metabolism like COL2A1 and ACAN were highly expressed in NP cells, together with highly expressed SOX9 gene (25, 29); PDGFRA and PRRX1 were mainly expressed in NP progenitor cells (NPPCs), which were the two mesenchymal progenitor markers (28); stromal cells highly expressed COL1A1 and COL3A1 genes (27); endothelial cells mainly included specific marker genes of CDH5 and PECAM1 (49); erythroblast cells consist of CA1, HBB, GYPA, ALAS2, and AHSP; in terms of immune cells, monocytes/macrophages were identified by CD14, CD163, CD86 and MRC1 (50); B cells were identified with MS4A1 and IGKC (49); NK/T cells were identified with TRAC, TRBC2 and SPOCK2; neutrophils mainly expressed FCGR3B and CXCL8 (51); granulocytic myeloid-derived suppressor cells (G-MDSC) highly expressed marker genes of ITGAM and ARG1 (52, 53); and granulocyte macrophage progenitor (GMP) cells were identified by marker genes of ELANE, MPO, and MS4A3 (54, 55). The distribution of distinct cell populations between different degenerative stages is shown in **Figures 4H, I**.

### Oxidative stress levels among different cell populations of IDD

**Figure 5:**
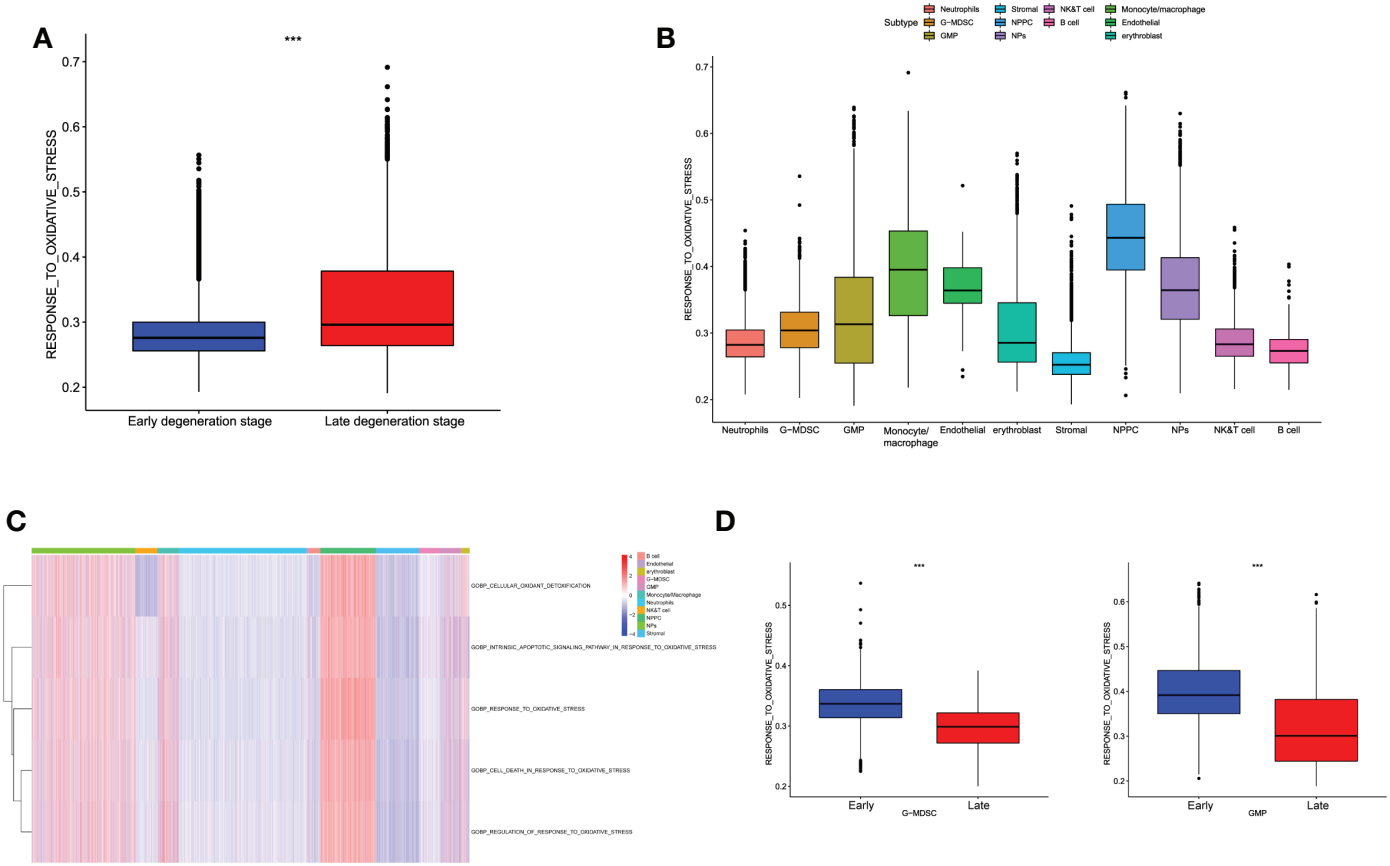
**(A)** Differences in cellular oxidative stress response of different groups at the single-cell level. **(B)** The OS-related functions among different cell populations. **(C)** The heatmap shows each OS-related function among different cell populations. **(D)** Comparison of OS-related functions between early and late stage in G-MDSC and GMP.

Based on the classified cell populations, we also performed “GSVA” algorithm to calculate the scores of OS-related functions for each single cell and observed the OS levels in real IVD microenvironment. The results of single cells in IDD were consistent with the bulk-RNA seq, in that the OS-related functions in late degenerative IVD tissues were significantly higher than that in early degenerative tissues (**Figure 5A**). Moreover, we evaluated the OS-related functions for each cell subtype. Results illustrated that the NP and NPPC cells had the highest OS-related scores. As for immune cells, monocytes/macrophages were shown as the most correlated immune cells with OS-related functions. Intriguingly, we also observed high OS levels in endothelial cells (**Figures 5B, C**). We next observed the OS-related functions among different cell subtypes between early and late degenerative IDD. Results suggested that most cell populations had higher OS-related levels in late degenerative IVD tissues except GMP and G-MDSC, as shown in **Figure 5D** and **Supplementary Figure S2**, which may behave protective roles to ameliorate IDD progression, at least by reducing OS levels.

### Sequential alterations of monocyte/macrophage populations during IDD

**Figure 6:**
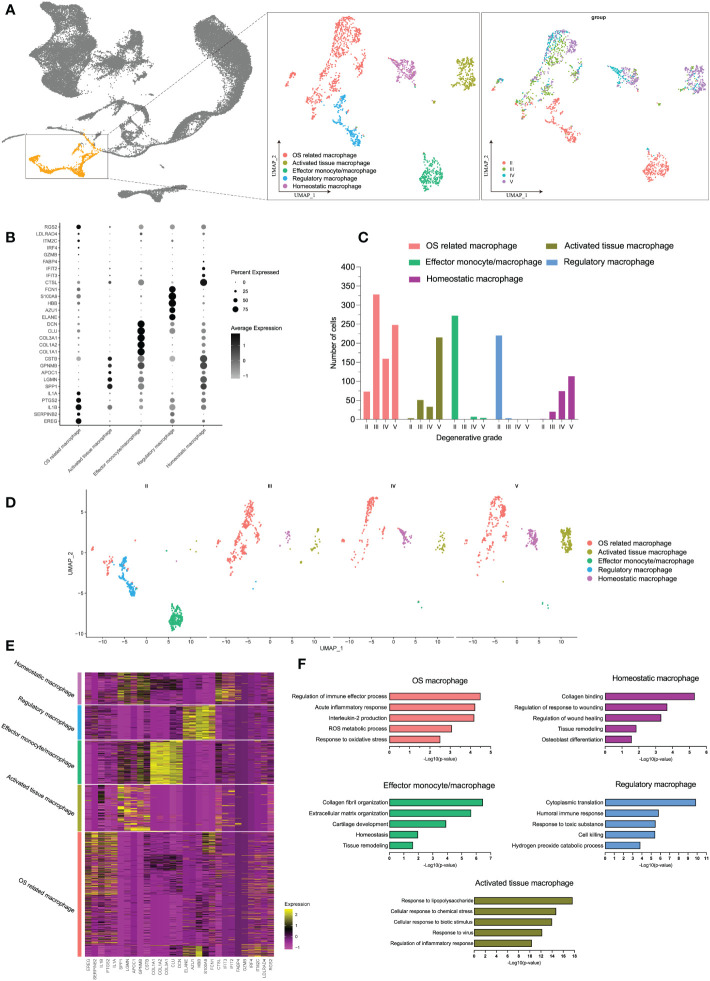
**(A)** The distribution of macrophages among all cell populations and the further re-clustering of macrophages with UMAP visualization. **(B)** Five different macrophage subtypes and their specific marker gene expression levels, with brightness indicating log-normalized average expression, and circle size representing the percent expressed. **(C)** The number cells of macrophage subtypes in different degeneration stage. **(D)** UMAP visualization of macrophage subtypes in different degeneration stage. **(E)** Heatmap of the top 5 of cluster-specific DEGs for each macrophage subtype; the color indicated the relative gene expression. **(F)** Bar plots showing the −log10 (p-value) from enrichment analysis of representative GO biological functions among different macrophage subtypes.

In immune cells, monocytes/macrophages possessed the highest OS-related functions in degenerative IVD tissues, whether by bulk-RNA-seq or scRNA-seq analysis. We next analyzed monocyte/macrophage-specific features that dynamically changed during IDD. Therefore, we extracted and performed re-clustering analysis of the monocyte/macrophage population through UMAP dimensional reduction method. Totally, we analyzed 1,824 monocyte/macrophage cells based on variable genes and identified six different sub-clusters (**Supplementary Figure S3A**). Based on the distinct gene signatures, we identified the following five monocyte/macrophage subtypes with distinct biological significance for downstream analysis: OS related macrophages (EREG^+^ IL1A^+^ macrophages), activated tissue macrophages (APOC1^+^ CLU^−^ macrophages), effector monocytes/macrophages (CTGF^+^ COL3A1^+^ monocytes/macrophages), regulatory macrophages (AZU1^+^ ELANE^+^ macrophages), and homeostatic macrophages (CTSL^+^ MRC1^+^ macrophages) (shown in **Figures 6A, B**; **Supplementary Figure S3B** **)**. **Supplementary Figure S3D** also displayed the normalized expression levels of representative marker genes for each subtype.

Although there were no significant number changes in monocytes/macrophages as a whole during early and late stages, we observed an interesting trend of subtypes after monocytes/macrophages re-clustering. The proportion of each subtype underwent distinct changes during IDD: the subpopulation of effector monocytes/macrophages and regulatory macrophages was dominant at grade II but was drastically decreased from grade III; with the progression of degenerative grade, we observed increased proportions of OS related macrophages, activated tissue macrophages, and homeostatic macrophages, while effector monocytes/macrophages and regulatory macrophages barely existed (**Figures 6C, D**). Dynamic changes in the proportions of the subtypes are summarized on UMAP plot in **Supplementary Figure S3C**.

To characterize the subtypes of monocytes/macrophages in detail, we identified DEGs for each subtype, and the top 50 DEGs of each subtype were analyzed by GO biological functions (**Figure 6E**). DEGs of OS-related macrophages (the dominant population in late degenerative IVD tissues) were enriched in functions like “acute inflammatory response,” “ROS metabolic process,” and “response to oxidative stress”; besides, they mainly expressed markers of M1 (IL1A, IL1B, TLR4, and IL6), which were considered to act roles like M1. Activated tissue macrophages had DEGs that were mainly associated with activated inflammatory response like “response to lipopolysaccharide” and “regulation of inflammatory response.” As expected, DEGs of homeostatic macrophages and effector monocytes/macrophages were chiefly enriched in protective functions like “tissue remodeling” and “collagen binding;” meanwhile, both two subtypes highly expressed M2 markers (CSF1R, MRC1, C1QB, and C1QC), which may behave roles like M2, and effector monocytes/macrophages also highly expressed monocytes marker (CD14) and proliferative markers (CTGF and PCNA) (56, 57). As for regulatory macrophages, they were mainly involved in functions of regulating effects, such as “cytoplasmic translation” and “humoral immune response” (**Figure 6F**; **Supplementary Figure S3D**). Overall, we totally defined five distinct subtypes of monocytes/macrophages, which suggested extensive heterogeneity with each other in different degenerative stages of IDD.

### Evolution lineage trajectory of different monocyte/macrophage subtypes in IDD

**Figure 7:**
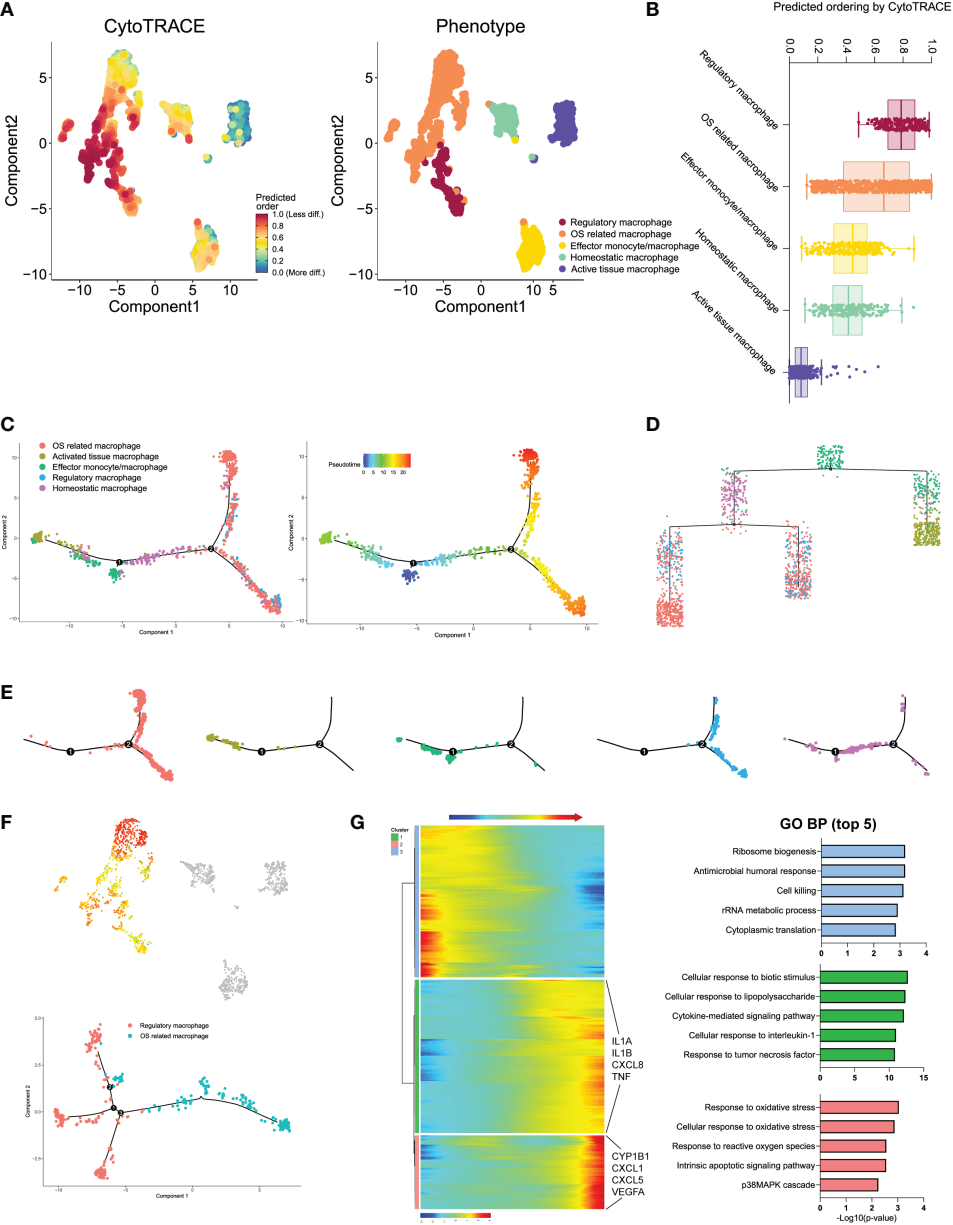
**(A)** Plot of the CytoTRACE pseudo-time order for the macrophage subtypes. **(B)** Calculation of CytoTRACE scores of each macrophage cell according to predicted order. **(C)** Monocle pseudo-time analysis revealed the macrophage subtypes lineage progression. The left panel displays the different macrophage subtypes along lineage progression; the right panel indicates the pseudo-time results of these subtypes. **(D)** Tree branch diagram of the macrophage subtypes; the legend of macrophage subtypes is the same as in panel **(C)**. **(E)** The detailed location of different macrophage subtypes along the lineage progression. **(F)** Pseudo-time trajectory initiated from regulatory macrophages toward OS-related macrophages. **(G)** The left panel shows the alterations of relative expression patterns of representative genes along the pseudo time. The right panel analyzed the GO biological functions in clusters 1–3, as defined in the left panel.

We next performed the CytoTRACE analysis to further evaluate the evolutionary dynamics of the monocyte/macrophage subtypes, which could reveal the direction of evolution and predict cell lineage trajectories using gene counts and expression (43). Few kinetics were observed in activated tissue macrophages, while regulatory and effector monocytes/macrophages formed complex kinetics during IDD (**Figure 7A**). To quantify the kinetic dynamics of CytoTRACE results, we calculated the scores of each cell in **Figure 7B**, which represent the evolutionary ability. Regulatory macrophages had the highest levels of dynamics. In contrast, activated tissue macrophages and homeostatic macrophages both had low levels of dynamics, which were consistent with the findings from UMAP embedding (shown in **Figure 7A**). The results suggested that regulatory and effector monocytes/macrophages contributed to the formation of the rest macrophages during IDD.

To investigated the dynamic transcriptome changes of these subtypes, we next constructed monocle pseudo-time trajectory analysis. The trace displayed that effector monocytes/macrophages that accumulated at the root of the trajectory, homeostatic, and activated tissue macrophages were distributed alongside different branches from the root, while regulatory and OS-related macrophages accumulated together at the tail end of the track (**Figures 7C, E**). The pseudo-time tree branch diagram further demonstrated the detailed evolution process of these monocyte/macrophage subtypes (**Figure 7D**). Results illustrated that effector monocytes/macrophages were located at the father node, which further divided into homeostatic and activated tissue macrophages. Regulatory and OS-related macrophages clustered together at the end of child nodes, which were consistent with the trajectory evolution results. These data indicated that separate trajectories of these subtypes were useful to depict their distinct evolution pathways; effector monocytes/macrophages could evolve into homeostatic and activated tissue macrophages, while regulatory macrophages could evolve into OS-related macrophages.

For the trajectory towards OS-related macrophages (OS route) (**Figure 7F**), we defined three distinctive clusters showing different modular gene expression alterations. We summarized the top 5 GO biological functions in **Figure 7G**; notably, clusters 1 and 2 of the OS-related macrophages route (which was exclusively expressed in highly activated OS-related macrophages) suggested increased expression of IL1B and IFN. In addition, highly activated OS-related macrophage clusters displayed predominant enrichment of pro-inflammatory mediators like IL1A, IL1B, CXCL8, and TNF (**Supplementary Table S2**). These findings pointed that the specific subpopulation that derived from regulatory macrophages had experienced IDD-dependent activation of macrophage inflammatory responses.

**Figure 8:**
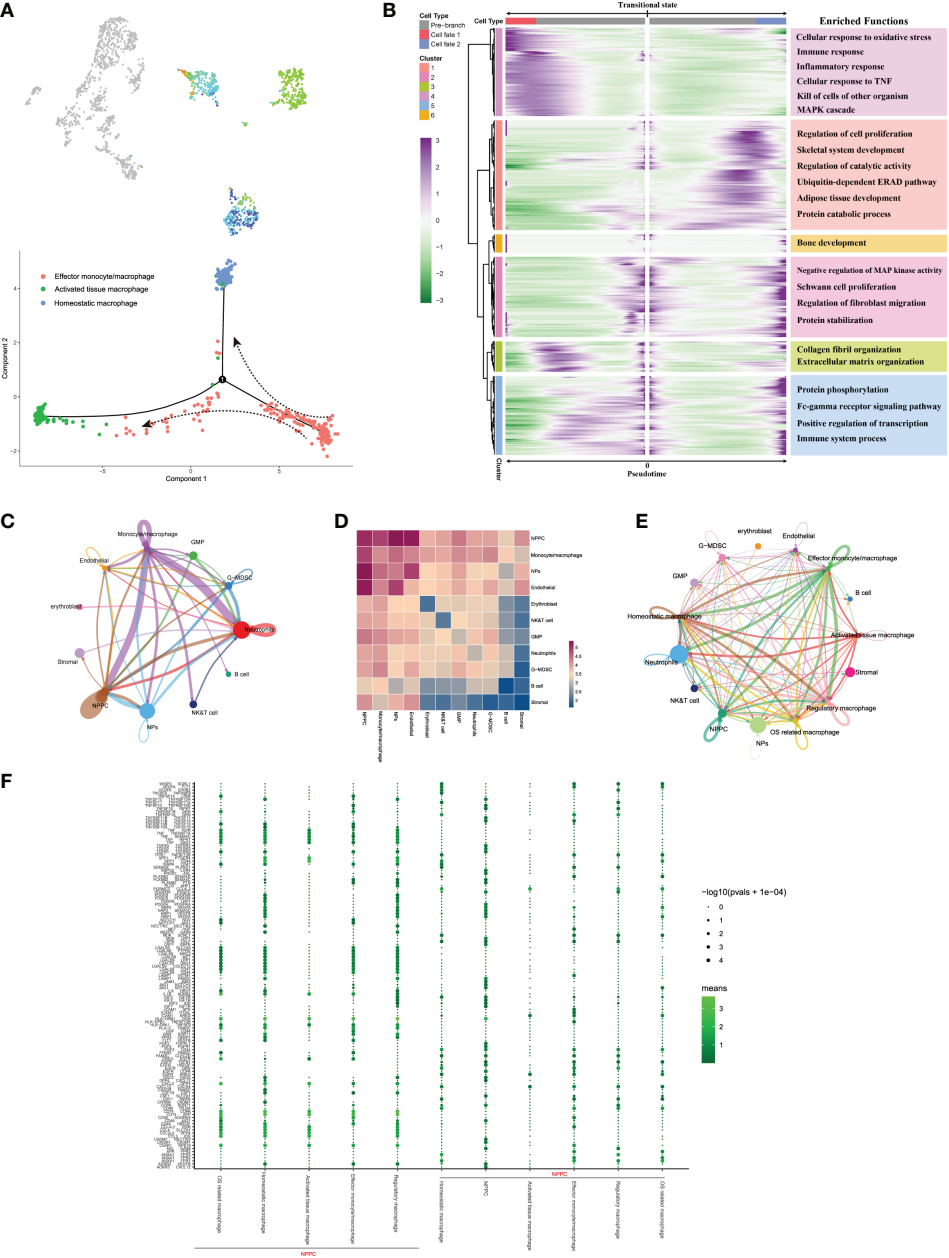
**(A)** Pseudo-time trajectory initiated from effector monocytes/macrophages toward activated tissue and homeostatic macrophages. **(B)** Branch trajectory heatmap of the DEGs revealed gene expression alterations under different lineage progression directions, together with the main functions of these genes in each cluster displayed. **(C–E)** Overview of the cellular inter-regulatory network and the correlation among different cell populations. **(F)** The bubble plot shows each statistically significant ligand–receptor pair between NPPC and macrophage subtypes.

As for the trajectory from effector monocytes/macrophages, we performed BEAM analysis for branch fate-determined gene analysis according to node 1, branch heatmap visualized significant gene expression alterations in different subtypes, cell type I represented the smaller state ID (namely activated tissue macrophages), and cell type II indicated bigger state ID (namely, homeostatic macrophages), as shown in **Figures 8A, B**. We totally defined six distinct clusters and analyzed their biological functions. Results suggested that genes in cluster 4 became highly expressed in activated tissue macrophages, with their biological functions associating with immune response, including cellular response to oxidative response and inflammatory response. In contrast, genes in the rest clusters were mainly overexpressed in homeostatic macrophages, with their biological enrichment with protective functions like regulation of cell proliferation, collagen fibril organization, and extracellular matrix organization; they also showed increased expression of C1QA and TGFB2, which were known to be key genes of well-differentiated M2 macrophages (42).

In brief, the monocyte/macrophage subpopulations experienced time-dependent and subtypes-specific alterations during IDD progression. These subpopulations exhibited a continuous spectrum changes at the transcriptome level, which mainly began with effector monocyte/macrophages and regulatory macrophages from early degenerative stage.

### Signaling network for the intercellular crosstalk regulating the homeostasis of NP Cells

To seek further insights about the critical factors and the pathways that contribute to the activation or homeostasis of NP cells, especially between different monocyte/macrophage subtypes and NP cells, we investigated the signaling network in the whole cell populations based on CellphoneDB and Cellchat analysis. Results displayed highly regulated cellular communications from the bidirectional interactions among those cell populations; the more and thicker lines indicated more participated signaling pathways and more interaction weights, and the size of circles represented the numbers of each cell population (**Figure 8C**). Communication correlation heatmap quantified the numbers or weights among different cell populations, which demonstrated that the NPPC, NPs, and monocytes/macrophages had actively regulated roles (**Figure 8D**); thus, monocytes/macrophages were determined as the essential immune niche components in degenerative IVD tissues. We further divided monocytes/macrophages into distinct subtypes, and regulatory network indicated that each subtype had distinct active interactions with other cell populations (**Figure 8E**). The detailed signaling communication results between different subtypes and other cell populations are shown in **Figure 8F**.

**Figure 9:**
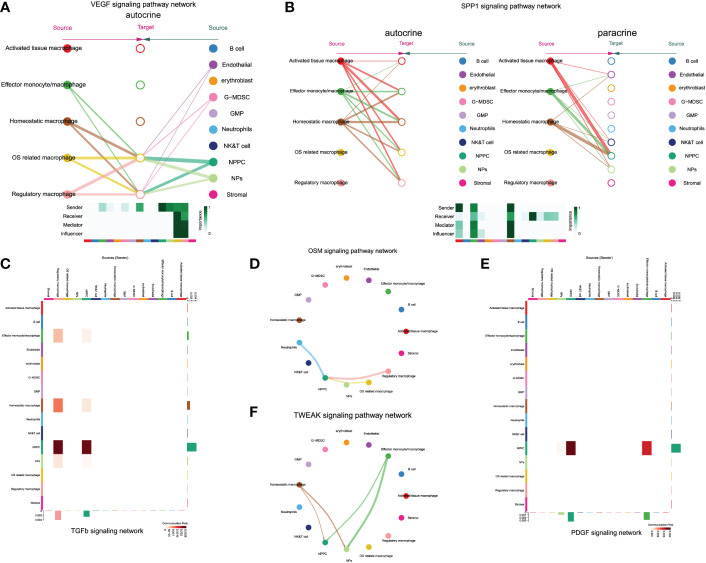
The enrichment situation of VEGF **(A)**, SPP1 **(B)**, TGFβ **(C)**, OSM **(D)**, TWEAK **(E)** and PDGF **(F)** signaling pathways respectively.

Notably, only regulatory and OS-related macrophages were involved in vascular endothelial growth factor (VEGF) signaling pathway through autocrine, which also served as receiver of VEGF signals, while NPs and NPPC functioned as the main regulators of the communication (**Figure 9A**). The SPP1 pathway had significant changes in NPPC, NP cells, NK/T cells, and endothelial cells, which were especially participated by monocytes/macrophages (except regulatory and OS-related macrophages subtypes) (**Figure 9B**). Moreover, the tumor growth factor beta (TGF-β) pathway was modulated by regulatory macrophages and NPPC, mainly through autocrine or paracrine mediation by TGFB1-TGFBR1 or TGFB1-ACVR1 interactions. Intriguingly, the OSM signaling pathway was mainly observed in regulatory and OS-related macrophages through paracrine, targeting NPPC. In the platelet-derived growth factor (PDGF) signaling network, the effector monocytes/macrophages acted as critical contributions by secreting PDGFA, PDGFB, and PDGFC ligands, leading to the paracrine from effector monocytes/macrophages to NPPC and NP cells. In addition, the TWEAK signaling pathway was mainly mediated by effector monocytes/macrophages and homeostatic macrophages through paracrine targeting NPPC and NP cells; the detailed information of these activated signaling pathways is shown in **Figures 9C–F**. These findings could also advance the intervention targeting potential signaling pathways to ameliorate IDD.

### Transcription factors regulatory network among monocytes/macrophage subtypes

**Figure 10:**
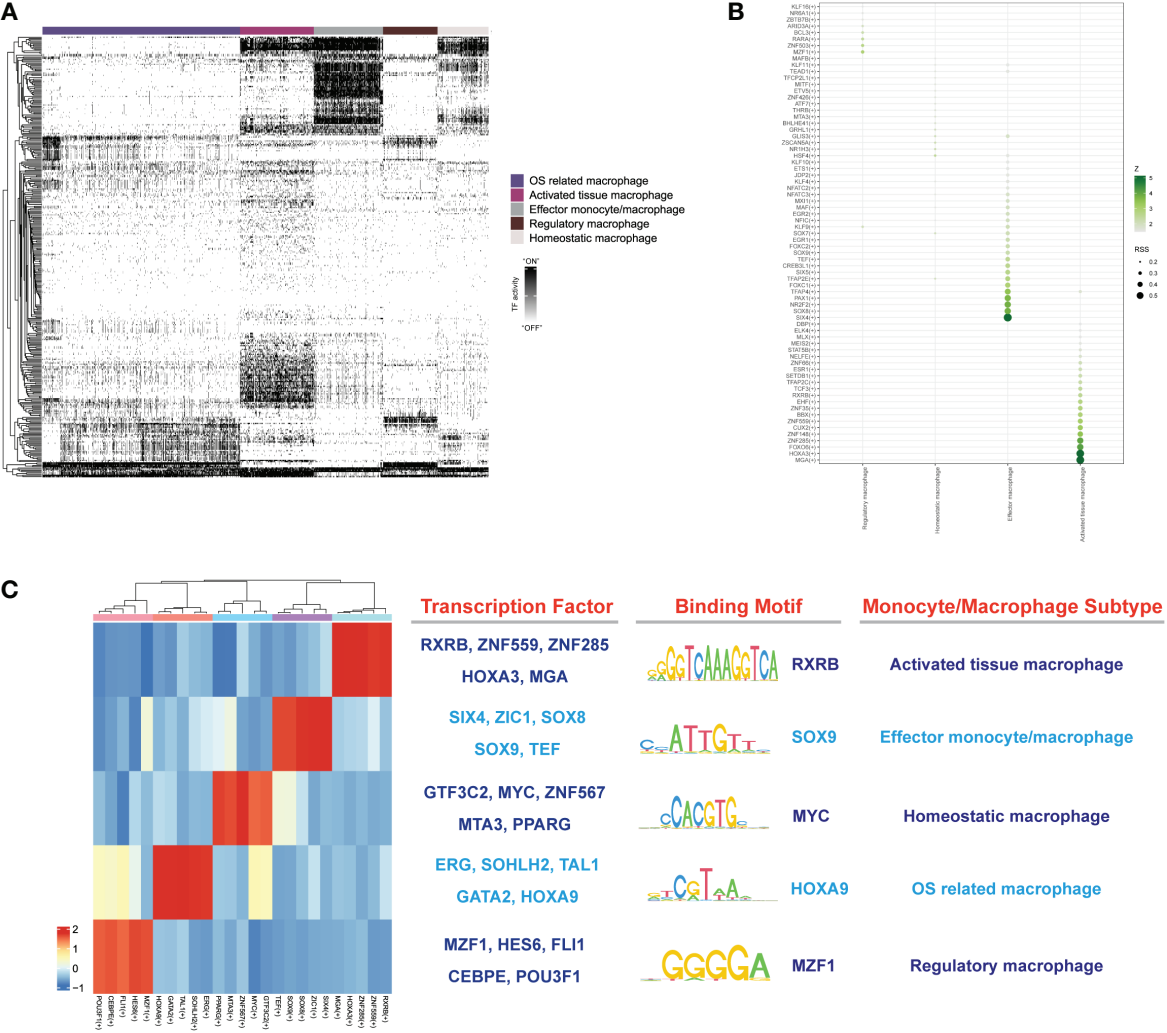
**(A)** The heatmap reveals binary regulon activities analyzed with SCENIC in each subtype of macrophage; “ON” indicated active regulons and “OFF” indicated inactive regulons. **(B)** Hub TFs calculated by calcRSS algorithm. **(C)** The heatmap displays the identified regulon modules for each macrophage subtype, along with representative transcription factors, corresponding binding motifs, and associated cell types.

To explore the gene regulatory networks that determined cell fate in the monocyte/macrophage subtypes, we performed SCENIC analysis to decipher the regulatory activity (regulons) from the co-expression of transcription factors (TFs). Totally, we discovered 357 regulons that were used to discriminate the different monocytes/macrophage subtypes (**Figure 10A**). After the calculation of regulon specificity score (RSS), totally, 25 regulons were further determined as the core regulons that modulate the downstream functions. As shown in **Figures 10B, C**, results illustrated that different subtypes upregulated the expression of different TF regulatory networks. The highly enriched regulons in activated macrophages included RXRB, HOXA3, ZNF559, ZNF285 and MGA. The SIX4, SOX8, SOX9, ZIC1, and TEF TFs were specific to effector monocytes/macrophages. Homeostatic macrophages exhibited strong enrichment of MTA3, GTF3C2, MYC, ZNF567, and peroxisome proliferator- activated receptor gamma (PPARG) TFs. OS-related macrophages were enriched in regulons such as ERG, GATA2, HOXA9, TAL1, and SOHLH2. As for regulatory macrophages, they mainly overexpressed MZF1, HES6 CEBPE, FLI1, and POU3F1 TFs. The representative motifs of each monocyte/macrophage subtype are illustrated in **Figure 10C**. These identified TFs helped researchers further realize the gene regulatory network by monocytes/macrophages and made it possible for cell therapies to mitigate the IDD.

## Discussion

The cellular heterogeneity of IVD tissues has been a long-debated controversy due to the complexity of IDD (28). Although recent studies have reported several single-cell transcriptomes of physiological or degenerative IVD tissues, none have used a longitudinal approach along with the natural disease course. Inflammatory responses are closely related to oxidative stress and immune processes (58), while few studies have focused on the aberrantly expressed biomarkers associated with immune infiltration and oxidative stress in degenerative IVD tissues.

Herein, based on the differential analysis, WGCNA, and immune infiltration results, we finally determined four hub genes, namely, CYP1A1, MMP1, CCND1, and NQO1, which suggested tight associations with the progression of IDD. The functional enrichment annotations of DEOSGs further helped us realize the potential mechanisms of IDD that oxidative stress responses, xenobiotic stimulus, ferroptosis, and metabolism of cytochrome P450 were mainly involved in the pathological processes of IDD. We previously discovered that lipid-metabolism gene CYP1B1 from blood tissues could promote the development of IDD through immune infiltration (59); this study further found that CYP1A1 behaved OS-related functions in IDD. The characteristic feature of cytochrome P450 (CYPs) enzyme could produce H_2_O_2_ directly or indirectly *via* superoxide anions, which were also known as “NADPH oxidase” activity. H_2_O_2_ produced by CYPs could lead to the accumulation of cytotoxic ROS, which thereby impairs cell functions and causes tissue damage (60). This is the first time that the essential roles of CYPs family with oxidative stress and immune responses were connected with the pathological process of IDD, which were worth of subsequent in-depth research. Existed studies have reported the essential roles of MMPs family and their inhibitors screening in the progression of IDD, among which MMP1 may participate partially through OS-related functions and inflammatory reactions (61, 62). NQO1 served as antioxidant genes and played essential roles in ameliorating inflammatory responses, reticulum stress, and apoptosis of degenerative NP cells, which were partially regulated by the antioxidant transcription factor Nrf2 (14, 63).

According to the immune infiltration analysis based on bluk-RNA seq, Tregs, plasma cells, and monocytes/macrophages were aberrantly activated in the progression of IDD. The results of the significant changes in immune cells by immune infiltration analysis could give us evidence in the following focused research by scRNA-seq. The immune infiltration analysis is a developed algorithm based on gene expression. The 22 immune cell types have already been determined since the algorithm was developed. Thus, single-cell results further verified the above findings that different immune cell infiltrations occurred in the early stage of IDD.

The number of neutrophils increased with the degenerative stages, which also played essential roles in OS-related functions. This study further intuitively demonstrated the importance of neutrophils infiltration in mediating the development of IDD from the single-cell level, which confirmed our previous deduction that neutrophils could arrive and accumulate in degenerative IVD through blood vessels and acted directly on NP cells by releasing inflammatory factors, thereby undermining the immune privilege of IVD (59). G-MDSC was widely defined as immature neutrophils, with immunosuppressive functions that inhibit T-cell activation and ROS production, which has been widely studied in neoplasms, acting as a suppressor of anti-tumor immune response (64–67). The numbers of G-MDSC were found to be decreased in late degenerative IVD, and the potential protective roles of G-MDSC have been reported by Tu et al. in IDD (25). This study further determined that this protective process may be achieved by reducing the levels of oxidative stress. In addition, GMP also possessed low levels of OS-related functions, and the numbers were found to be decreased in late degenerative IVD, which may behave similar protective roles with immunosuppressive functions to ameliorate IDD as G-MDSC did. Moreover, we observed that endothelial cells also possessed high OS scores except monocytes/macrophages in IDD. Endothelial cells are vascular, non-conventional immune cells that participated in angiogenesis, homeostasis, and the regulation of vascular tone; they are also essential and active components of immune responses (68, 69). Existing studies have reported the OS functions of endothelial cells in different diseases like sepsis, acute inflammation, and neurovascular defects of the retina, and that OS activated endothelial cells, changed multiple endothelial cells functions, and promoted pro-inflammatory, pro-coagulant, and pro-adhesive phenotype (70, 71). Our findings further indicated the essential connections between endothelial cells and the progression of IDD on OS levels.

The inaccurate classification of monocytes/macrophages subtypes by immune infiltration analysis resulted in the module difference in WGCNA network. Based on the high levels of OS-related functions of monocytes/macrophages in degenerative IVD, we further extracted monocytes/macrophages to explore the longitudinal alterations of distinct phenotypes during the different degenerative stages. Monocytes/macrophages are heterogeneous, and their phenotypes and functions are regulated by the surrounding microenvironment. The detailed classification of monocytes/macrophages could help better understand the different roles in the progression of diseases. Lee et al. have conducted longitudinal approach based on scRNA-seq to reveal the sequential changes of macrophages during severe acute respiratory syndrome coronavirus 2 (SARS-CoV-2) infection, which classified nine distinct macrophage subtypes and exhibited exciting results in the progression of SARS-CoV-2 infection (42). In our study, we have provided the evidence that monocytes/macrophages infiltration occurred in the early degenerative stage of IVD. Importantly, we depicted the longitudinal alterations of monocytes/macrophages and NP cells heterogeneity within degenerative IVD tissue based on single-cell level. We discovered and annotated five different subpopulations among monocytes/macrophages, with their sequential and subtypes alterations from early to late degenerative IVD. There remained complex monocyte/macrophage subtypes in early degenerative IVD, with different pro- or anti-inflammation functions. We also observed distinctive and stepwise evolution lineage among those subtypes. The predominant dynamic changes in the transcriptome mainly involved effector monocytes/macrophages and regulatory macrophages, which drastically decreased at degenerative grade III, suggesting that these populations experienced shift of transcriptome features towards other populations. Based on the CytoTRACE and pseudo-time trajectory analysis, we could infer that OS-related macrophage clusters were derived from regulatory macrophage clusters, which also indicated that OS-related macrophages increased with degenerative stages, while with insufficient infiltration at early stage. The effector monocytes/macrophages could further shift into two subtypes of homeostatic and activated tissue macrophages with distinctive functions, demonstrating that although inflammation-related macrophages increased in severe degenerative IVD, protective macrophages also existed against inflammation.

Increasing studies have reported not only that blood cells are composed of endothelial cells but also that vascular-like structures could be generated by non-endothelial cells (18, 72). Macrophages are reported to regulate blood vessels growth, the new blood vessels could serve as passage for macrophages into ECM, and the interactions between macrophages and NP cells are potentially proangiogenic in degenerative IVD (73–76). Notably, Barnett et al. have demonstrated the significant finding that macrophages could form vascular mimicry channels in tumors or other hypoxic environments the same as cancer stem cells did (77). In intercellular crosstalk signaling network, we found that regulatory and OS-related macrophages mainly participated in VEGF signaling pathway, which may behave similar functions in degenerative IVD tissues, to form vascular mimicry channels themselves. The identification of this novel type of channels may help understand the development of IDD. Targeted clearance of regulatory macrophages from early stage and OS-related macrophages from late stage could reduce the angiogenesis. In addition, we also analyzed and reported other regulatory pathways between different monocyte/macrophage subtypes and NPPC or NP cells. These potential signaling pathways may provide personalized treatment targeting different subtypes through certain interactions.

Finally, a group of specific TFs were identified with distinct functions in different monocyte/macrophage subtypes, together with their corresponding binding motifs. Some TFs were reported to behave different functions to modulate the homeostasis of NP cells. SOX9 played essential roles in chondrocytes differentiation. Existing study demonstrated that the deletion of SOX9 could cause severe IDD characterized by apoptosis and matrix remodeling (78, 79). PPARG was reported to regulate osteoclast and osteoblast differentiation. Baroi et al. have already reported its essential roles in the treatment of osteoporosis (80). MTA3 is a latest identified member in the MTA family. It usually downregulates the expression of SNAIL, a major regulator of epithelium–mesenchymal transition, and subsequently inhibits the invasion and migration of tumor cells (81). More relationships between MTA3 and IDD are worth discovering. Zhang et al. have confirmed the negative correlations of HOXA9 and IDD, in which HOXA9 played pivotal roles in FAS-mediated apoptosis of NP cells and HOXA9 could serve as potential therapeutic target in the treatment of IDD (82). FLI1 belongs to the Ets transcription factor family and is highly expressed in activated immune cells, which modulates the development of monocytes/macrophages by the C-terminal transcriptional activation domain. In addition, FLI1 also participated in affecting the function of immune cells by regulating cytokines and chemokines (83, 84). These identified TFs made it possible for cell therapies to mitigate the IDD. Through the TFs, we can definitely advance the development of NP cell homeostasis by combining the potential TFs and downstream therapeutic targets in specific monocyte/macrophage subtypes, within the degenerative IVD microenvironment.

Collectively, this study combined transcriptome and single-cell sequencing results to systematically decipher the difference in OS-related functions of different cell populations within degenerative IVD tissues and further depicted the longitudinal alterations of immune cells, especially monocytes/macrophages in different degenerative stages of IDD. These findings further enhanced the understanding of the distinct functions of monocyte/macrophage subtypes during IDD progression. This study proposed that specific therapy strategies need to be formulated on different stages of IDD, thereby providing personalized treatment based on specific monocyte/macrophage subtypes.

## Data availability statement

The original contributions presented in the study are included in the article/**Supplementary Material**. Further inquiries can be directed to the corresponding authors.

## Author contributions

This study was completed with teamwork. Each author had made corresponding contribution to the study. Conceived the idea: WL, MY, BG, and ZL. Manuscript draft: WL, and YZ. Technical support on analysis: WL, YZ, ZH and CL. Downloaded and collected data: WL, YZ, BG, MY, ZH, LZ and BY. Analyzed the data: WL, YZ, BG, MY, and ZL. Prepared figures: WL, YZ, ZH, LZ, and YW. Redressed the manuscript: all authors. All authors contributed to the article and approved the submitted version.

## Supplementary material

Supplementary Figure 1
**(A)** PCA heatmap illustrated expression patterns of the top 30 significantly correlated genes in each component. Colors represented gene expression levels and PC_1 to PC_10 were displayed. **(B)** Dot plots illustrated the significantly correlated genes in each component, and PC_1 to PC_4 were displayed. **(C)** Red dots represented highly variable genes and black dots represented non-variable genes. The top 10 most variable genes were marked. **(D)** UMAP plot displayed the dot distribution after samples integration. **(E)** The expression components situation of each immune cell. **(F)** The enrichment fraction of 22 types of immune infiltrating cells in each sample.

Supplementary Figure 2 The Comparison of OS-related functions between early and late stage in different cell populations.

Supplementary Figure 3
**(A)** UMAP plot of macrophage subtypes, colored according to clusters. **(B)** Proportion of each macrophage population at different degeneration stages. **(C)** UMAP plot with color density reflecting distribution of macrophage subpopulations at different degeneration stages. **(D)** UMAP plot illustrating normalized expression of markers of macrophage subtypes.

Supplementary Table 1 The OS-related genes obtained from GeneCards database.

Supplementary Table 2 The DEGs that were identified for lineage progression from regulatory macrophage toward OS related macrophage.
