# Supplementary figures and images for: Deciphering the sequential changes of monocytes/macrophages in the progression of IDD with longitudinal approach using single-cell transcriptome
Source: Front Immunol. 2023 Feb 1;14:1090637. doi: 10.3389/fimmu.2023.1090637 (PMC9929188; doi:10.3389/fimmu.2023.1090637)

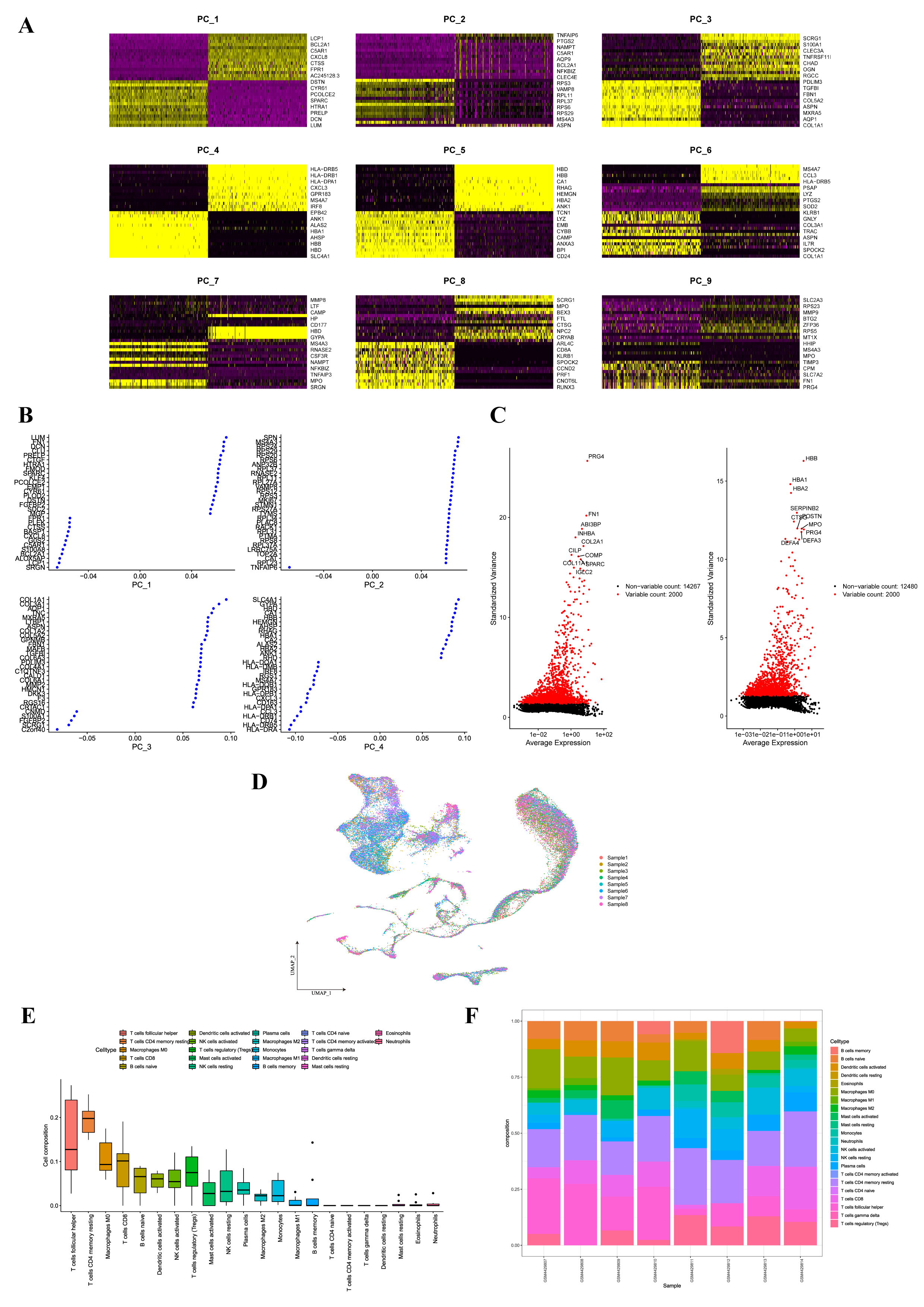

Supplement: Supplementary Figure 1 — (A) PCA heatmap illustrated expression patterns of the top 30 significantly correlated genes in each component. Colors represented gene expression levels and PC_1 to PC_10 were displayed. (B) Dot plots illustrated the significantly correlated genes in each component, and PC_1 to PC_4 were displayed. (C) Red dots represented highly variable genes and black dots represented non-variable genes. The top 10 most variable genes were marked. (D) UMAP plot displayed the dot distribution after samples integration. (E) The expression components situation of each immune cell. (F) The enrichment fraction of 22 types of immune infiltrating cells in each sample. [file Image_1.tif]

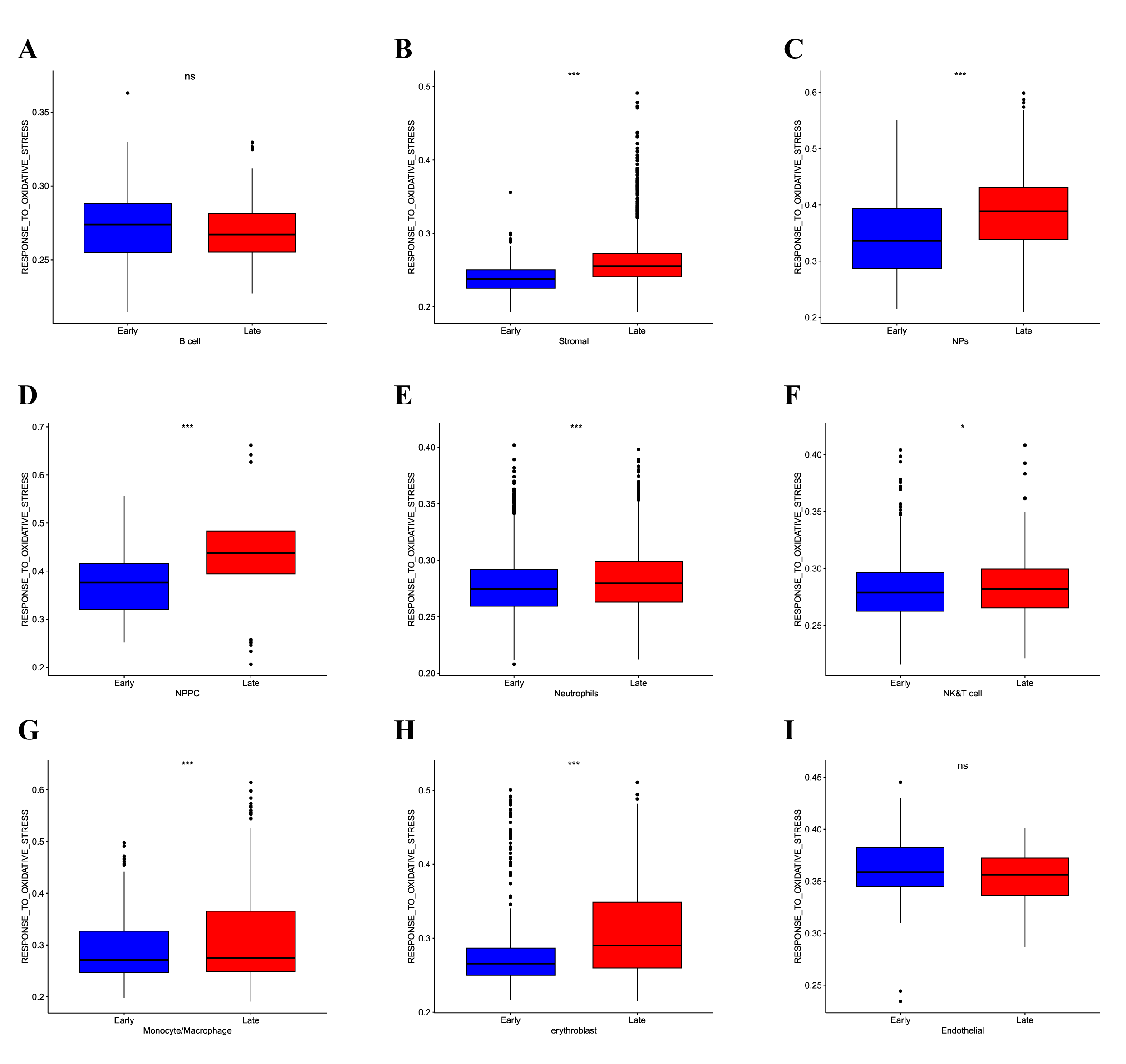

Supplement: Supplementary Figure 2 — The Comparison of OS-related functions between early and late stage in different cell populations. [file Image_2.tif]

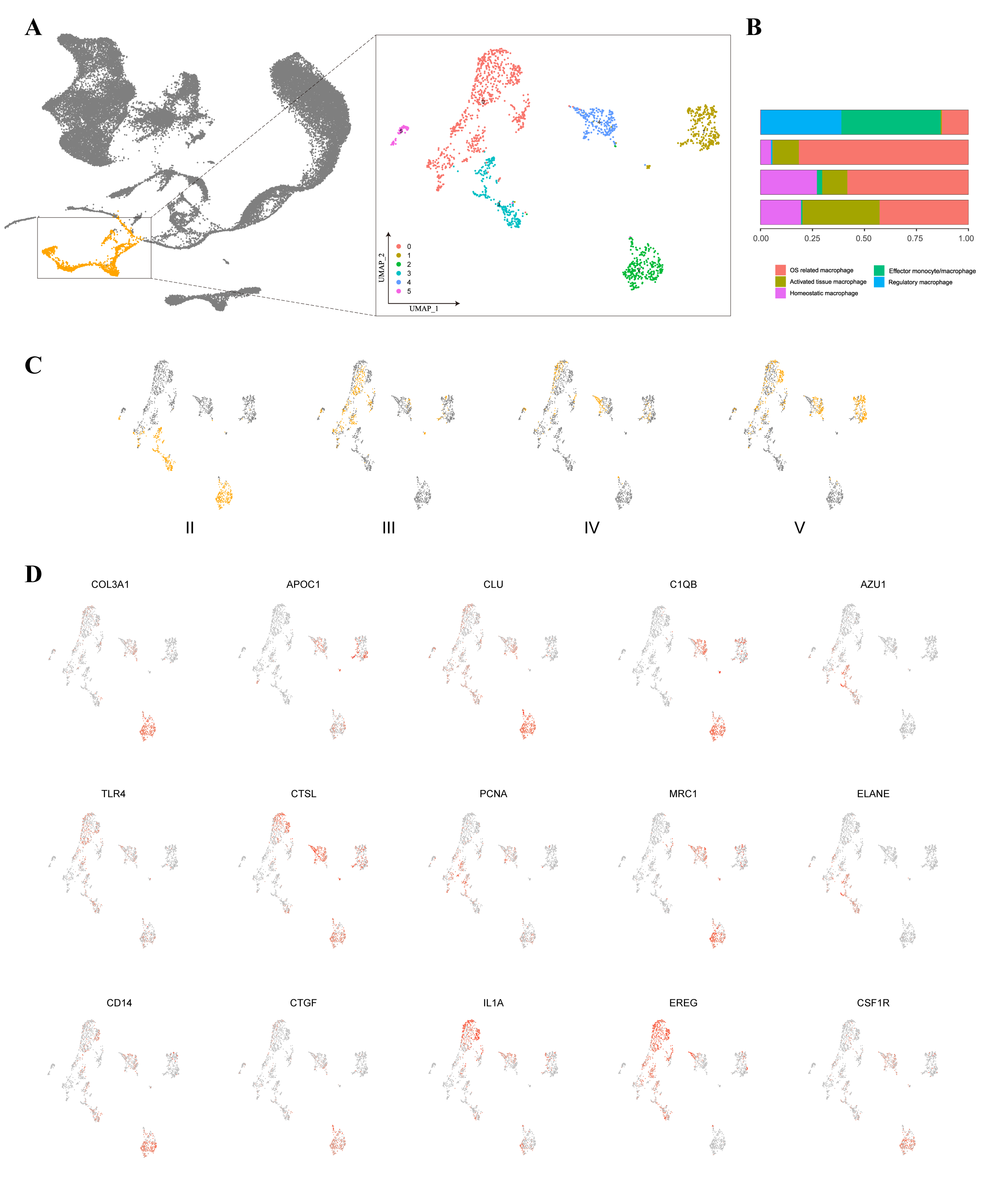

Supplement: Supplementary Figure 3 — (A) UMAP plot of macrophage subtypes, colored according to clusters. (B) Proportion of each macrophage population at different degeneration stages. (C) UMAP plot with color density reflecting distribution of macrophage subpopulations at different degeneration stages. (D) UMAP plot illustrating normalized expression of markers of macrophage subtypes. [file Image_3.tif]
